# Nutrients and Nutraceuticals from *Vitis vinifera* L. Pomace: Biological Activities, Valorization, and Potential Applications

**DOI:** 10.3390/nu17030583

**Published:** 2025-02-05

**Authors:** Cecilia Prata, Chiara Zalambani, Francesca Rossi, Simone Rossello, Teresa Cerchiara, Concettina Cappadone, Emil Malucelli

**Affiliations:** 1 Biochemistry Laboratory, Department of Pharmacy and Biotechnology, Alma Mater Studiorum University of Bologna, Via Irnerio 48, 40126 Bologna, Italy; cecilia.prata@unibo.it (C.P.); chiara.zalambani2@unibo.it (C.Z.); 2 Pharmaceutical Biochemistry Laboratory, Department of Pharmacy and Biotechnology, Alma Mater Studiorum University of Bologna, Via San Donato 19/2, 40127 Bologna, Italy; francesca.rossi105@unibo.it (F.R.); emil.malucelli@unibo.it (E.M.); 3 Drug Delivery Research Laboratory, Department of Pharmacy and Biotechnology, Alma Mater Studiorum University of Bologna, Via San Donato 19/2, 40127 Bologna, Italy; simone.rossello2@unibo.it

**Keywords:** grape pomace, polyphenols, fiber, bioactive compounds, biological activity, nutraceuticals, cosmetics, circular economy

## Abstract

Grape pomace, also known as wine pomace, is a by-product of winemaking that has traditionally been discarded. However, recent studies have highlighted its rich nutritional and bioactive potential, positioning it as a promising resource for various applications in the functional food, pharmaceutical, and cosmetic sectors. This review explores the nutrient and nutraceutical contents of grape pomace, including its high levels of polyphenols, dietary fiber, vitamins, minerals, and melatonin. The biological activities of grape pomace, such as antioxidant, anti-inflammatory, antimicrobial, and anticancer effects, are also discussed, emphasizing its potential as raw material endowed with multifunctional properties. Additionally, the valorization of grape pomace as a food supplement and for the development of cosmetics is examined, focusing on its incorporation into dietary products and skincare formulations. The growing interest in the sustainable utilization of grape pomace is underscored, highlighting its significant role in promoting human health and contributing to a circular economy.

## 1. Introduction

Agro-industrial waste poses a multifaceted challenge, intersecting economic, social, and environmental domains. The conventional ‘linear economic model’, based on the principles of extraction, production, and disposal, has been increasingly criticized for its unsustainable trajectory and the associated risks spanning societal and ecological spheres [1]. In response, the circular economy and bioeconomy frameworks emerge as transformative paradigms, offering an alternative to dismantle the ‘linear paradigm’. At their core lies the reduction, reuse, and recycling principles pivotal to sustainable waste management and the pursuit of long-term environmental resilience [2].

Innovative valorization strategies hold the potential to reframe food and agro-industrial waste as valuable resources, enabling the production of high-value derivatives and bridging the gap between waste generation and resource scarcity [3]. The integration of circular economy principles within agro-industrial systems not only mitigates waste management challenges but also catalyzes environmental, economic, and social benefits. These include significant reductions in environmental impact [4], lower operational costs through resource optimization [5], and the creation of employment opportunities, particularly in regional contexts [6].

The circular economy model has gained significant attention from academia, industry, and policymakers, as evidenced by the expanding research, rising scientific publications, and the focus of international organizations on the topic [7,8]. Despite these advances, significant obstacles persist. The scalability of circular models remains constrained by technical, economic, and regulatory barriers, necessitating targeted research to optimize bioprocesses and enhance systemic implementation [9]. Furthermore, societal factors often exert a more profound influence than technological challenges, as public acceptance and behavioral shifts are critical to the success of circular initiatives [10]. Consumer acceptance of products derived from circular economy practices is shaped by an interplay of personal values, contextual influences, and product-specific attributes [11].

One compelling example of circular economy principles in action is grape pomace (GP), a by-product of winemaking. Rich in bioactive compounds and nutrients, it exemplifies the potential to transform agro-industrial waste into value-added products across diverse fields [12].

**Figure 1:**
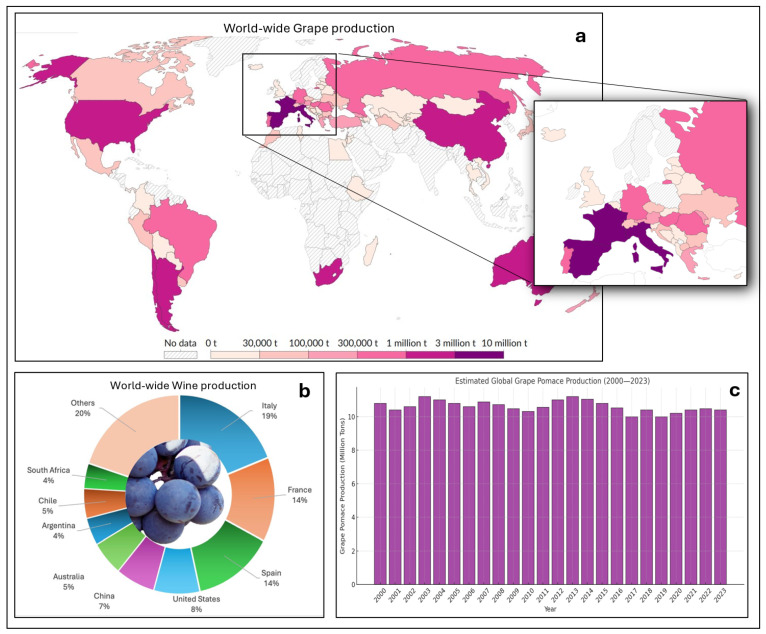
(**a**) World map chart illustrating grape production by geographic region (adapted from Food and Agriculture Organization of the United Nations (2023)—with major processing by Our World in Data); (**b**) Pie chart showing the countries with the highest wine production; (**c**) Histogram representing the estimated global grape pomace production from 2000 to 2023. The values are based on trends in global wine production and assume an average pomace yield of 25% of the total grape weight.

Worldwide, vineyards cover about 7.2 million hectares and the production from this area is around 77 million tons. The countries with the highest wine production in the world are Italy (5 Mt), France and Spain (3.7 Mt), USA, China, Australia, Chile, Argentina, and South Africa (Figure 1a,b). These countries together account for more than 80% of the world’s wine production, FAOSTAT: “https://www.fao.org/faostat/en/#data/QCL” (accessed on 13 January 2025). Among the agri-food sectors, the wine industry is responsible for the consumption of natural resources and for the generation of large amounts of solid and liquid residues to be discarded as waste [13,14].

Grape pomace, also known as grape marc or wine pomace, is a significant and degradable by-product of the winemaking process, representing about 20–25% of the total grape weight used in wine production [8,15].

This by-product consists of mixture of grape skins (43% of total grape pomace), seeds (23%), stems (25%), and pulp remnants [16,17].

Globally, around 8.49 million tons of grape pomace are produced [18]. Analyzing data from the International Organization of Vine and Wine (OIV), global wine production in 2023 was around 260 million hectoliters, which corresponds to approximately 40 million tons of processed grapes. Based on these numbers and considering an average yield of 25%, the production of grape pomace is around 10 million tons. Furthermore, analyzing the data since 2000, the quantity of grape pomace produced annually seems to remain almost constant (Figure 1c).

In major wine-producing countries like Italy, France, and Spain, annual grape pomace generation can reach nearly 1200 tons per year. The environmental impact of these by-products is significant, with pomace alone estimated to produce 834,000 tons of CO_2_eq in Italy based on 2016 wine production [19]. Considering the worldwide production of 292 million hectoliters of wine [20] estimated the emission, from the grape pomace, more than 4,776,776 tons of CO_2_.

In addition, the disposal of this by-product is one of the main concerns of the wine industry, as well as a significant additional cost. In fact, discarding GP in landfills causes several environmental issues [17], such as soil and groundwater contamination, the generation of unpleasant aromas, microbial contamination, the attraction of plant disease vector (insects and flies), and serious health risks to both aquatic ecosystems and humans [21].

Several studies have been carried out in the last decade to valorize GP via sustainable approaches, including energy production, biogas, animal feed, and the extraction of value-added products [22], but traditionally it has been used for producing wine alcohol, food colorings, and grape seed oil. However, this by-product is rich in valuable compounds such as anthocyanins, volatile organic compounds, fibers, proteins, minerals, vitamins, tannins, lipids, and lignocellulosic compounds, presenting opportunities for sustainable exploitation in various industries, including food, cosmetics, and pharmaceuticals [15,16,18,23], where it can serve as an antioxidant, fortifying agent, and source of nutraceuticals [24].

Despite the numerous scientific studies supporting the nutraceutical and biological properties of GP, the integrated and multidisciplinary understanding of these aspects remains limited. In this review, we aim to critically analyze the recent existing literature, with particular attention to the biological effects of grape pomace extracts, including their antioxidant, anti-inflammatory, antimicrobial, and anticancer activities. We will also explore their technological potential for applications in the food and cosmetic industries. Additionally, we provide an overview of recent patents in the field, illustrating how these innovations are shaping the future of sustainable and functional applications. By offering a comprehensive and up-to-date analysis, we hope to promote a more informed and innovative use of this wine by-product, emphasizing its value as a sustainable resource for future advancements and contributing to the principles of circular economy.

## 2. Nutrients in *Vitis vinifera* L. Pomace

**Figure 2:**
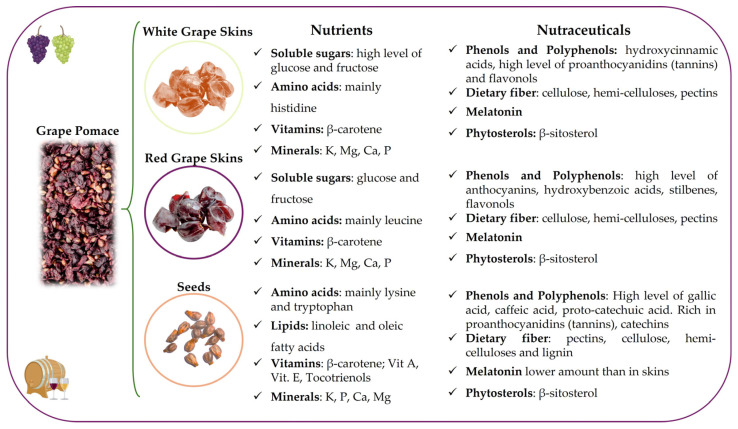
Nutrients and nutraceuticals present in white and red grape skins and in seeds.

Wine pomace has recently been studied as a potential source of bioactive compounds, aiming to enhance its value. The amount of pomace and its chemical composition, in terms of nutrients and nutraceuticals (summarized in the Graphical Abstract and detailed in Figure 2), are influenced by grape varieties, growth conditions (cultivation, climate, and soil), and winemaking processing practices used for wine production [25]. In particular, different grape varieties exhibit distinct profiles of nutrients and secondary metabolites (nutraceuticals/bioactive compounds), due to inherent genetic differences. Soil conditions, including its mineral content, pH, and water retention capacity, can influence grapevine growth and the synthesis of bioactive compounds. Environmental factors such as climate, altitude, and sunlight exposure further contribute to variations in the accumulation of these compounds. Additionally, the winemaking process, encompassing steps such as fermentation, maceration, and aging, can alter the chemical composition of the final product by influencing the extraction, transformation, and stabilization of bioactive compounds. These factors collectively determine the nutrient and bioactive compound composition of grapes, leading to significant differences across varieties and production methods.

Therefore, a range of concentrations of these compounds in pomace are here reported.

### 2.1. Soluble Sugars

White GP generally contains a high concentration of soluble sugars (70–107 g/kg), predominantly glucose and fructose, which account for over 78% of the total soluble sugars in GP. The total sugar content in red GP is usually in the range of 8–15 g/kg. GP typically has a higher concentration of these sugars compared to red GP due to differences in the wine-making processes. White GP is obtained immediately after the pressing of fresh grapes, retaining a significant quantity of soluble sugars. In contrast, red GP is derived from the separation and pressing of grape mash after maceration and alcoholic fermentation, during which *Saccharomyces cerevisiae* converts most of the soluble sugars into ethanol. The high content of soluble sugars in white GP can be recovered and utilized in various applications, such as the production of aldonic acids for the cosmetics and plastic industries or as substrates for aerobic or anaerobic fermentation processes to produce biofuels [26,27].

### 2.2. Amino Acids

The nitrogen content in wine grapes is crucial for yeast metabolism and growth, significantly impacting both grape and wine quality [28]. However, there are only a few studies that have characterized the amino acid content of by-products from the wine industry. Recently, it has been reported that the most abundant amino acid in red skins is leucine (3.1 g/100 g), while that in white skins is histidine (1.9 g/100 g) [29]. Several studies report the total amino acid content in seed extracts, in the range 5–20 g/100 [30,31], particularly rich in essential amino acids such as lysine. There is no quantitative difference in alanine concentration between red and white seed extracts [29].

### 2.3. Lipids

The lipid composition of wine pomace is primarily derived from the seeds and skins, with the seeds containing the highest concentration of lipids. The total lipid content in wine pomace generally ranges from 5% to 15% of dry weight, with variations depending on the grape variety, wine production methods, and the extraction process used. The lipid content in seeds may reach up to 15% of their dry weight, while skins generally have less than 7% total lipids, independently from red or white grapes [27].

In particular, *V. vinifera* seeds includes 90% of unsaturated fatty acids, mainly constituted by linoleic fatty acid (65–75%) and oleic fatty acid (20–40%), and 10% saturated fatty acids [32,33].

Therefore, the fatty acid profile of GP (especially seeds) is rich in unsaturated fatty acids, particularly PUFAs like linoleic acid and oleic acid. This high content of unsaturated fatty acids makes grape pomace a valuable source of plant-based oils with potential health benefits [34,35,36].

### 2.4. Vitamins

The vitamin composition of grape pomace varies depending on the grape cultivar and on the stability of the vitamins. For example, Vitamin C is present in grapes, but it is rapidly oxidized during the subsequent processes. However, some studies reported a vitamin C concentration that varied from 46.0 to 179.2 mg/1 kg of grape pomace [36]. The B group vitamins are found in low amounts in grape pomace.

Regarding lipophilic vitamins, Goufo et al. [37] found 50 mg of vitamin E exists per 100 g of grape seed, together with tocotrienols (499–1575 mg/1000 g); however, other studies have suggested different contents in the range of 86–244 mg alfa-Tocopherol/100 g [36].

The concentration of the antioxidant and precursor of vitamin A (β-carotene) was re-ported to be between 33.9 and 59.8 ppm in the seed oil of grape, together with other carotenoids, such as lutein [36]. Grape skins tend to contain the highest levels of carotenoids, including lutein, particularly in varieties with higher pigmentation. Although these carotenoids do not have pro-vitaminic activity, they contribute to the antioxidant power of grape pomace, particularly against singlet oxygen, which is the ROS associated with damage from UV radiation exposure [38].

### 2.5. Minerals

The amount of minerals in GP is non-negligible, suggesting a potential application in health supplements and cosmetics [39,40]. As previously stated, the composition of GP can vary based on the grape variety, geographical location, and the winemaking process. However, according to different scientific reviews, the main minerals found in this by-product GP are here listed and briefly discussed.

Potassium (K), an essential micronutrient for regulating fluid balance, muscle contractions, and nerve function [41], is abundant in GP (1–9 g/kg). Potassium bitartrate (KHC_4_H_4_O₆), or cream of tartar, is a by-product that commonly forms in wine production and is found in GP, which is the solid residue left after the pressing of grapes. Cream of tartar (potassium bitartrate) in grape pomace is typically not present in copious quantities; however, it can be extracted and used in culinary applications (such as in baking and stabilizing egg whites), as well as in some industrial processes.

The concentration of phosphorus (P), essential for energy production and maintaining healthy bones and teeth [42], generally ranges from 0.5–3 g/kg in GP.

Magnesium (Mg) is typically found in GP with a concentration around 0.3–2 g/kg. It plays a vital role in enzyme function, muscle and nerve function, and in the synthesis of proteins [43].

Grape pomace contains also a significant amount (200–500 mg/kg) of calcium (Ca), which is a micromineral important for bone and teeth, besides being involved in contraction of muscles and in nerve functioning, blood clotting, blood pressure regulation, and the immune system [44].

As described in a recent article, GP is an excellent source of minerals that can be used for enriching the nutritional value of food and feed [45].

## 3. Nutraceuticals in *Vitis vinifera* L. Pomace

Nutraceutical is a term coined by Stephen De Felice in 1989 and is derived from the two words “nutrition” and “pharmaceutical”. It refers to “any substance that is a food or a part of a food and provides medical or health benefits, including the prevention and treatment of chronic degenerative diseases”, as reported in “Scientific Concepts of Functional Foods in Europe Consensus Document” [46].

(Poly)phenolic compounds and dietary fiber, two of the most widely recognized dietary factors contributing to human health [47,48], are considered the most important classes of nutraceuticals of wine pomace [27,49].

### 3.1. Phenolic Acids and Polyphenols

Although variables such as cultivation method, temperature, soil conditions, grape variety, geographical origin, and winemaking methods can strongly influence the chemical composition of GP [50], GP represents a valuable source of phenolic acids, stilbenes, and other different members of the polyphenol family.

Total polyphenol content can vary over a wide range of 0.28–8.70 g/100 g of the sample. Polyphenolic compounds of GP are mainly represented by hydroxybenzoic (gallic, ellagic, vanillic, syringic, and *p*-hydroxybenzoic acids) and hydroxycinnamic (ferulic, caffeic, and *p*-coumaric acids) acids; flavonols (kaempferol, myricetin, and quercetin and their derivatives); flavanols (catechin, epicatechin, gallocatechin, epigallocatechin, and epicatechin 3-O-gallate); anthocyanins (cyanidin, delphinidin, petunidin, peonidin, and malvidin and their 3-glucoside derivates); and non-flavonoids (tannins, stilbenes, coumarins, and neolignans) [27,51,52,53,54,55].

The extraction yield of grape polyphenols typically ranges from 30% to 40%, influenced by factors such as grape variety, vineyard location, and the technological parameters of wine production, including destemming, crushing, pressing, and maceration. Specifically, grapes contain 60–70% of their total phenolic content in the seeds and 28–35% in the skins [37].

In another recent study, the variable composition of polyphenolic compounds in skin and seed extracts from GP, derived from white and red winemaking of different Italian grape varieties, was assessed. A recent study examined the variable composition of polyphenolic compounds in skin and seed extracts from GP derived from both white and red winemaking of different Italian grape varieties. The study measured the total polyphenolic content (expressed as mg gallic acid equivalent (GAE) per gram of dry weight), the main classes of polyphenolic compounds, and the DPPH index. It was found that seed extracts consistently contained higher levels of total polyphenols and condensed tannins, as well as exhibiting stronger antiradical activity compared to skin extracts: 144–298 mg GAE/g d.w. extract for skins and 327–540 mg GAE/g for seeds; the DPPH values were 1.77–3.40 mg AAE/g for skins and 3.10–10.48 mg AAE/g for seeds [56].

Grapes contain a range of phenolic acids, present in both free and conjugated forms, with hydroxybenzoic and hydroxycinnamic acids being the most prevalent. The hydroxybenzoic acids in grapes include 4-hydroxybenzoic, gallic, protocatechuic, syringic, and vanillic acids, while the main hydroxycinnamic acids are caffeic, *p*-coumaric, ferulic, and synaptic acids [57].

Moreover, grape polyphenol concentrations vary significantly depending on the grape fraction. The highest total phenolic content is observed in grape seeds, followed by grape skin and pulp. Specifically, the total polyphenolic content in grape seeds is reported to be 130 times greater than in grape pulp (GAE). The polyphenolic profile of grapes is intricate, with 78 phenolic compounds tentatively identified in grape samples using LC-ESI-QTOF-MS/MS, as outlined in a recent review [58]. The flavonoids typically present in grapes include flavan-3-ols (flavanols), flavonols, and anthocyanins. Proanthocyanidins, which are the most abundant form of flavan-3-ols in grapes, consist of proanthocyanidin monomers, oligomers, and more complex polymers. Among these, the four major proanthocyanidins are (+)-catechin, epicatechin, procyanidin B1, and procyanidin B2.

Flavonols, which accumulate predominantly in grape skin, can also be present in the seeds of certain grape varieties; notable flavonols include quercetin, myricetin, and kaempferol. All these molecules are known to play an important role in counteracting oxidative stress, inflammation, and related pathologies [59].

Anthocyanins, water-soluble pigments, are responsible for color and for plant defense and are primarily concentrated in the skin of pigmented grape cultivars, such as red or purple grapes, while they are absent in white grape varieties. Clinical evidence suggests that anthocyanins can enhance endothelial function and vascular health in individuals with cardiovascular diseases. The protective role of anthocyanins is primarily related to vascular endothelium, being associated with improvements in microcirculation. These effects have positive implications for counteracting cardiovascular diseases and enhancing vision and cognitive functions [60,61].

Proanthocyanidins are the primary form of condensed tannins in grape seeds. These tannins are potent antioxidants due to their ability to scavenge free radicals and inhibit oxidative damage to cells. Tannins are responsible for the astringent taste of GP. This characteristic is particularly prominent in the seeds and red skins, where tannins can bind to proteins in the mouth, causing a dry sensation. Further, it is well known that tannins are endowed also with anti-inflammatory, antimicrobial, and anticancer properties [62,63].

The combination of flavonoids, catechins, procyanidins, phenolic acids, and tannins in *Vitis vinifera* seeds and skin contributes to their strong antioxidant activity by means of synergistic multi-target activity. This activity is primarily due to the following mechanisms: direct free radical scavenging, metal chelation that prevents the copper- and iron-dependent formation of harmful free radicals, and indirect antioxidant activities linked to the modulation of gene expressions [64]. In addition to antioxidant powers, they are known to play important anti-inflammatory and antimicrobial roles important in preventing and counteracting chronic degenerative diseases [65].

Among stilbenes, another large class of plant secondary metabolites belonging to polyphenols, piceatannol is present in grape; however, resveratrol (3,5,40-trihydroxystilbene) is the most common, mainly in the *trans* form. Red grape skin is a well-known source of resveratrol, a phytoalexin that, like other phenolic compounds, serves as a defense mechanism against environmental stress and pathogenic threats, including fungal infections, injury, and UV radiation [66]. Recently, a role played by grape pomace stilbenes as anti-obesity agents has been recently discussed [67].

Several recent reviews show the comparison between different composition in polyphenols of grape skin and seeds of different white and red wine waste, as summarized in Table 1.

### 3.2. Dietary Fiber

Grape pomace is a considerable source of dietary fiber, with grape skins serving as the primary contributor [68]. The fiber content is influenced by grape variety, with red grape skins comprising 51–56% by weight, whereas white grape skins contain 17–28% [2]. The majority of dietary fibers in grape skins consist of insoluble fibers, including cellulose, hemicelluloses, and lignin [15]. These insoluble fibers are characterized by high porosity and low density, which enhance gastrointestinal efficiency by modulating nutrient absorption, increasing fecal bulk, and promoting peristalsis [69]. Some fiber compounds in GP make chemical bonds with phenolic substances and, thus, create antioxidant dietary fibers, giving the pomace stronger radical scavenging potential. Furthermore, certain fiber compounds in grape pomace form chemical interactions with phenolic compounds, resulting in antioxidant-enriched dietary fibers. This synergy confers a higher radical scavenging capacity to the pomace, thus elevating its nutritional value relative to dietary fibers in cereals. Research has demonstrated the beneficial effects of these complex fiber–phenolic associations on human health [70].

Pectin, a soluble polysaccharidic fiber, is mostly found in GP in the skin and to a lesser extent in the seeds. It is part of the complex structure of the fruit’s cell wall, where it plays a role in providing structure and rigidity. The concentration of pectin in GP can vary depending on factors like the grape variety and winemaking process, but it is generally considered to be significant. Studies suggest that GP can contain anywhere from 5% to 10% pectin by weight [71]. Pectin has various health benefits, including supporting digestion, lowering cholesterol levels, and promoting gut health and it is also used as a gelling agent, stabilizer, and thickener in jams, jellies, and marmalades.

In *Vitis vinifera* L. grape seeds, the total content in fiber is about 40.2–43.7 g per 100 g [32,36].

### 3.3. Melatonin

Melatonin (N-acetyl-5-methoxytryptamine), an indole-based structure (MW 232.28), is ubiquitous in living organisms, although melatonin was considered exclusively as an animal neurohormone for a long period of time. Starting from 1995, melatonin has been identified in many different plants in a wide range of concentrations (from picograms to micrograms per gram of tissue), defined as phytomelatonin [72].

This metabolite was first identified in grapes in 2006 as a secondary metabolite produced via the shikimate pathway, a seven-step metabolic process utilized by bacteria, archaea, fungi, algae, certain protozoans, and plants to synthesize folates and aromatic amino acids. Melatonin has been detected in various cultivars of *Vitis vinifera* L. at concentrations ranging from 0.005 to 440.0 µg/kg dry weight [73].

As recently reported, winery by-products displayed the following (significantly different at *p* < 0.05) decreasing order of melatonin concentration: GP (0.902 µg/kg dw) > wine lees (0.586 µg/kg dw) > grape stems (0.234 µg/kg dw) [74].

Numerous evidence indicates that melatonin is involved in the regulation of circadian rhythm and influences both antioxidant enzyme activity and cellular mRNA levels of antioxidant enzymes. Melatonin improves mitochondrial respiration and increases ATP synthesis under physiological and inflammatory conditions [75,76]. Arnao and Hernández-Ruiz suggest the use of phytomelatonin as a potential nutraceutical [77].

The relatively high content of melatonin in GP deserves attention by scientific and industrial fields, as recently suggested [78].

### 3.4. Phytosterols

Phytosterols (also known as plant sterols) are plant-derived compounds that have structural similarities to cholesterol and have gained attention for their health benefits, particularly in lowering cholesterol levels and improving cardiovascular health [79]. β-sitosterol is the most abundant phytosterol in GP and is the major bioactive component contributing to its cholesterol-lowering properties. Other sterols like campesterol and stigmasterol are present in smaller amounts but contribute to the overall phytosterol profile. In this context, Harbeoui et al. investigated the unsaponifiable fraction of seed oil from three grape varieties (Merlot, Carignan, and Syrah), identifying the presence of two triterpene compounds (mirein and lanosterol) and six phytosterols (campesterol, D7-avenasterol, stigmasterol, β-sitosterol, β-sitostanol, and cholesterol) [80].

Further studies are needed to optimize the extraction and bioavailability of phytosterols from GP to maximize its health benefits.

The rich variety of nutrients and nutraceuticals present in GP, summarized in Figure 2, highlights the high potential value of what, until a few years ago, was considered a waste product.

## 4. Extraction Methods for Bioactive Compounds from *Vitis vinifera* L. Pomace

Currently, traditional solvent extraction or green extraction methods can be used to recover bioactive ingredients from agricultural wastes, including GP. The selection of solvents and the application of heat and/or agitation are crucial factors of the traditional solvent extraction process [15]. According to Drosou et al. [81], the suitable solvents for recovering phenolic compounds from GP include water, water: ethanol (1:1), and ethanol. They are characterized by different polarities to favor the solubility of different bioactive compounds. Although the extraction yields of the extracts are strongly dependent on the nature of the solvent and the plant’s moisture content, the extraction technique affects the recovery of polyphenols too. The most widely used traditional extraction techniques are solid-liquid extraction by mechanical agitation, maceration, and Soxhlet extraction.

These methods are characterized by low equipment costs, simple flowcharts, and high yields [82]; but they require long extraction times, abundant solvent consumption, and evaporation or concentration process after extraction to obtain purer bioactive compounds. Considering these disadvantages, green extraction technologies, for example, supercritical fluid extraction (SFE), microwave-assisted extraction (MAE), ultrasound-assisted extraction (UAE), pulsed electric field (PEF), and enzyme-assisted extraction (EAE), are currently studied to extract bioactive ingredients from wine industry waste [18,83]. These technologies are more efficient due to their reduction of the extraction time, energy consumption, cost, or quantity of solvent, ensuring high quality and safe extracts. Detailed overview on the principles of extraction methods of bioactive compounds by GP were reviewed by Wang et al. [2] and Castellanos-Gallos et al. [18]. In addition, several authors have studied the recovery of polyphenols from pomace using the aforementioned extraction techniques. Casas et al. [84], Da Porto et al. [85], and Farías-Campomanes et al. [86] have successfully used SFE, and in particular supercritical carbon dioxide (SC-CO_2_), to recover polyphenols from GP. Caldas et al. [87] has specifically used MAE for recovering quercetin, rutin, catechin, and epicatechin as the main phenolic compounds in grape skins. In addition, anthocyanins and generally heat-sensitive bioactive compounds may be extracted by UAE, as reported by Da Porto et al. [88] and Drosou et al. [81]. Brianceau et al. [89] has demonstrated that PEF is a selective process for the recovery of anthocyanins from GP.

Recently, an innovative green solvent extraction method based on natural deep eutectic solvents (NaDESs) has garnered interest for the extraction of GP compounds, especially polyphenols [2,18,90]. NaDESs are prepared by mixing hydrogen-bond donors (HBDs) and hydrogen-bond acceptors (HBAs) at an appropriate molar ratio to make a eutectic mixture [91,92]. Jeong et al. [93] used a mixture of citric acid (HBA) and maltose (HBD) in a 4:1 M ratio as a NaDES to successfully extract anthocyanins from grape skin. The comparison of anthocyanin extraction efficiency using this NaDES with traditional methanol and ethanol extraction methods revealed that the NaDES extraction was more than twice as efficient. Panic et al. [94] investigated anthocyanin NaDES extraction from GP on a larger scale. Firstly, the research group selected the appropriate NaDES mixtures, optimizing the extraction parameters, and then investigated solvent recycling. Specifically, the anthocyanin recovery was almost 90% and the NaDES recycling yield was 77.91%. Although the utilization of renewable resources and low energy requirements has made NaDESs an appealing green alternative for extracting bioactive chemicals, their toxicity profiles differ greatly depending on their composition. Therefore, as required by regulations for food and pharmaceutical uses, toxicological studies must be conducted to determine safety for human health and environmental exposure [95].

## 5. Biological Activities

The biological activities of GP have been extensively studied, revealing its considerable influence on human health and its versatile potential for diverse applications. As emphasized in the introduction, targeted investigations into the health benefits of by-products present a promising pathway for applied research, aligning with the principles of a circular economy aimed at reducing food waste and its associated environmental and economic repercussions.

The four most well-characterized biological properties of *Vitis vinifera* L. pomace are its antioxidant, anti-inflammatory, antimicrobial, and antiproliferative activities. Although the interplay between oxidative stress and the pathophysiological mechanism of inflammation is increasingly understood, the antioxidant and anti-inflammatory effects are discussed in separate sections to enhance clarity and readability. This delineation is supported by the substantial body of literature specifically addressing the anti-inflammatory properties of GP as reported in Table 2. This table provides a comprehensive overview of grape varieties investigated for their biological activities, summarizing findings from both in vitro and in vivo studies on GP extracts.

### 5.1. Antioxidant Activity

Free radicals, particularly ROS such as superoxide (O2^•−^), hydroxyl (OH^•^), peroxyl (RO_2_^•^), and hydrogen peroxide (H_2_O_2_), are naturally produced during metabolism, inflammation, and other physiological processes. While ROS play crucial roles in functions like intracellular signaling, cell proliferation, phagocytosis, and apoptosis, their excessive production can lead to oxidative stress. This condition is associated with damage to macromolecules such as lipids, proteins, or DNA, determining cell damage, immune dysfunction, and fatigue. To counteract this, living organisms rely on antioxidant mechanisms—both enzymatic and non-enzymatic, such as catalase or glutathione (GSH). Furthermore, dietary antioxidant supplementation has been shown to effectively mitigate the harmful effects of oxidative stress on human health.

In this context, GP extracts can be of the utmost importance, due to their capability of reducing oxidative stress. Goutzourelas et al. demonstrated that grape pomace (GP) extracts, rich in polyphenols and derived from wine production by-products including peels, seeds, and stems, attenuated oxidative stress in muscle and endothelial cells, primarily by increasing glutathione (GSH) levels [96]. Moreover, a follow-up study evaluates the induction of two key enzymes involved in GSH metabolism: γ-glutamyl cysteine synthetase and glutathione S-transferase. The results highlight the pivotal role of GSH systems in mediating the antioxidant properties of GP extracts, which emerges as a promising candidate for use as a dietary supplement aimed at managing oxidative stress-related disorders in the cardiovascular and skeletal muscle systems [97].

Some authors have explored the antioxidant and cardioprotective properties of GP extract obtained from a red *Vitis vinifera* L. variety grown in Romania. Both fresh and fermented extracts from this grape variety exhibited substantial antioxidant activity in vitro and in vivo. Remarkably, in vivo investigations revealed that the extract confers cardioprotective effects in isoproterenol-induced myocardial ischemia, primarily by mitigating oxidative stress [98]. Among the cardioprotective properties, one notable benefit associated with the ethanol extract of winery by-products is its strong inhibitory effect on platelet aggregation. This finding highlights the potential for utilizing GP in the development of functional foods with cardioprotective benefits [99].

In the development and progression of cardiovascular diseases endothelial cells are integral to the regulation and coordination of signaling processes within the vascular wall. Therefore, human umbilical vein endothelial cells (HUVECs) are widely utilized as a model to investigate vascular responses to natural antioxidant therapies. Posadino et al. demonstrated that GP extract exhibits significant antioxidant activity coupled with vasculo-protective effects. It has also shown the capability to mitigate H_2_O_2_-induced oxidative stress and prevent cell death in primary human endothelial cells [100]. Others have demonstrated that the anthocyanins in Pinot Noir pomace extract can influence target genes associated with the Nrf2 signaling pathway in endothelial cells. Molecular docking analysis revealed that among the anthocyanins, cyanidin-3-glucoside, malvidin-3-glucoside, and peonidin-3-glucoside exhibit the strongest affinities for binding to Keap1. This suggests that these compounds may alter the interaction between Keap1 and Nrf2, potentially affecting the pathway’s regulatory mechanisms [101].

The antioxidant activity of Taurisolo^®^, a novel nutraceutical formulation derived from winemaking by-products of the Aglianico cultivar, was assessed in human immune cells. Taurisolo^®^ consists of a grape polyphenol extract microencapsulated in maltodextrins to enhance polyphenol bioavailability. Notably, it significantly reduced ROS levels and improved intracellular antioxidant enzyme systems in neutrophils [102].

The antioxidant potential of optimized extracts derived from winery by-products produced in Brazil’s semi-arid region was assessed. The investigation focused on the capacity to neutralize radical species, alongside evaluating cytotoxicity and the release of TNF-α in the RAW 264.7 macrophage cell line. In particular, the inactivation of radical and non-radical ROS, such as hypochlorous acid (HOCl), was also observed. HOCl is a highly reactive species that is naturally generated in neutrophils by myeloperoxidase, an enzyme responsible for catalyzing the oxidation of chloride ions by H_2_O_2_. Although HOCl is involved in defense mechanisms against microorganisms ingested by neutrophils, its excessive production can lead to significant tissue damage [103].

Erythrocytes serve as an effective model for assessing the antioxidant efficacy of natural bioactive compounds, facilitating a better understanding of their biological radical-scavenging properties. Manconi et al. verified that bioactive compounds derived from red GP incorporated into liposomes exerted antioxidant activity in erythrocytes from healthy donors [104]. Additionally, other authors demonstrated that Mexican GP extracts exhibited antioxidant activity on erythrocytes. The extracts effectively reduced membrane anisotropy caused by radical inducers, restoring it to levels comparable to untreated cells and preserving membrane fluidity [105].

The antioxidative effect of polymer-associated liposomes loaded with GP extract has been established in intestinal cells, suggesting a potential application in the nutraceutical field. The ability to restore healthy conditions after hydrogen peroxide-induced stress in Caco-2 cells was demonstrated [106]. The effects of GP on the gut have also been investigated using animal models. Specifically, the administration of polyphenols from GP was evaluated in the duodenum and colon of piglets fed a diet containing 5% GP. A significant reduction in lipid peroxidation and enhanced antioxidant status were observed. Additionally, superoxide dismutase activity significantly increased in the duodenum, while catalase and glutathione peroxidase activities were markedly elevated in the colon [107].

Another interesting study was conducted on the human intestinal cell line Caco-2 to evaluate the efficacy of nutriosomes, grape extract-loaded fiber-enriched vesicles. These were prepared by combining antioxidant extracts obtained from *Vitis vinifera* var. Cannonau cultivar pomace and a prebiotic soluble fiber. The formulations were found to be highly biocompatible and able to protect cells from oxidative stress damage after exposure to H_2_O_2_. The treatment with the GP extract in aqueous solution reduced the damaging effect of H_2_O_2_ in stressed cells. When the extract was incorporated into vesicles, it enhanced the protective effect on the cells, preventing cell death and even promoting cell proliferation [108].

Furthermore, the GP extract proved to be protective on HL-60 leukemic cells under oxidative conditions conferred by the presence of H_2_O_2_. The extracts exhibited potent protective activities on cell growth and mitochondrial membrane potential (Δψm), comparable to the activity of the reference antioxidant compound Trolox [109].

Interestingly, extracts from red GP cultivars were shown to protect human hepatocytes in an in vitro experimental model of steatosis. Significant inhibition of fatty acid-induced lipid accumulation, modulation of redox processes through intracellular ROS production, and the inhibition and disruption of lipid peroxidation cascade reactions were observed [110].

A strong antiradical activity of GP obtained from Nero d’Avola grape, was described not only in Hep-G2 but also on human skin fibroblast HS-68. The seeds and skin of the GP were analyzed for their nutritional and antioxidant composition, fatty acid and polyphenols profile, and bioactive properties in vitro [111].

The grape skin sorted from the pomace of Carignano cultivar proved to be a suitable matrix to obtain a phytocomplex rich in bioactive compounds characterized by high antioxidant capability. Again, the ability of the extract to protect fibroblasts from oxidative stress was improved by its loading into liposomes, glycerosomes, and Montanov™-glycerosomes [112]. Additional research confirmed that encapsulation of the extracts optimizes the stability and scavenger capacity of the radicals. The ethanolic extract obtained from grape skins (Cabernet Sauvignon and Feteasca Neagra) were encapsulated in mesoporous silica matrices and tested on human keratinocytes (HaCat). Excellent cytocompatibility and, in some cases, stimulation of cell proliferation was recorded. Keratinocytes, together with fibroblasts, are a crucial cell type for the evaluation of natural antioxidants, given their clinical relevance and potential applications in cosmetics and nutraceuticals [113,114].

An interesting area of study for GP concerns the correlation between its antioxidant activity and its effect on cell differentiation. It has been demonstrated that GP extracts from Arneis and Croatina display interesting osteo-inductive properties that result in the activation of BMP2 and Runx2 gene expression [115]. Accordingly, other authors showed that functionalizing a nano-textured titanium surface with polyphenols can potentially enhance the osteogenic activity of osteo-integrated implants. In particular, the interaction of osteoblasts with titanium surfaces nano-functionalized with a grape pomace extract solution promotes the formation of a mineralized matrix [116].

The effects of GP extract on whole genome expression levels in a mouse myoblast cell line were investigated. GP appeared to influence cellular differentiation, as evidenced by the downregulation of the gene encoding cyclin B1, with its expression reduced by 50% [157].

The antioxidant activity of GP extracts exhibits a multifaceted mechanism of action, encompassing the upregulation of glutathione systems, modulation of the Nrf2-Keap1 signaling cascade, and direct neutralization of reactive oxygen species. This pleiotropic approach contributes to the extracts’ efficacy in mitigating oxidative stress across diverse cellular and tissue types. Additionally, the implementation of advanced delivery systems, such as liposomal and nutriosomal formulations, has demonstrated enhanced bioavailability and improved efficacy of these bioactive compounds, further expanding their potential therapeutic applications.

### 5.2. Anti-Inflammatory Activity

Inflammation is a multifaceted biological process triggered by harmful stimuli such as pathogens, damaged cells, or irritants. This response plays a crucial protective role by targeting the root cause of tissue injury, removing damaged cells, and facilitating the repair of affected tissues. It involves a coordinated effort by the immune system, including the activation of immune cells, the release of signaling molecules like cytokines and chemokines, and an increase in blood flow to the site of injury. While acute inflammation is essential for healing and defense, prolonged or chronic inflammation can lead to the onset and progression of several diseases, such as autoimmune disorders, cardiovascular conditions, neurological pathologies, and inflammatory bowel diseases (IBDs) [158].

IBDs are a significant public health concern, marked by rising incidence rates and a profound impact on quality of life. A recent study demonstrated that a symbiotic combination (i.e., a combination of probiotics and prebiotics) of GP extract and a *Lactobacilli* mixture exerts strong anti-inflammatory activities. This combination effectively downregulated numerous LPS-induced inflammatory genes, their associated proteins, and signaling molecules. Moreover, key pro-inflammatory cytokines and chemokines such as TNF-α, IFN-γ, IL-12p40, IL-7, GCSF, and GM-CSF were reduced to levels comparable to controls [120]. Further research highlights GP as a rich natural source of polyphenols with the potential to alleviate chronic gut inflammation while enhancing vascular endothelial function. In studies using Caco-2 intestinal epithelial cells grown either in monolayers or co-cultured with HMEC-1 endothelial cells, GP extract decreased, in a dose-dependent manner, the intestinal expression and release of inflammatory markers such as IL-6, MCP-1, MMP-9, and MMP-2, induced by LPS and TNF-α. Additionally, in tumor cells, GP inhibited the gene expression of other pro-inflammatory markers, including IL-1β, ICAM-1, VCAM-1, and COX-2. These anti-inflammatory effects were mediated through NF-κB inhibition [121].

The characterization of a set of GP samples from Uruguayan vineyards in terms of total phenolic content, antioxidant capacity, and anti-inflammatory activity has been evaluated in the human colon cancer cell line. The results indicate that Tannat GP has higher phenolic content and antioxidant capacity compared to Cabernet Franc. The anti-inflammatory properties were assessed through NF-κB modulation and IL-8 production [128].

Inflammation is widely recognized as a critical driver of neurodegenerative processes such as Parkinson’s disease and Alzheimer’s disease, both of which represent growing public health challenges. A hallmark of neurodegeneration is the activation of microglia—resident macrophages in the central nervous system that serve as the first responders to tissue damage or infection. While microglial activation is essential for tissue repair, excessive or chronic activation can exacerbate neurodegeneration through the release of pro-inflammatory and cytotoxic factors. This underscores the need to strike a balance between beneficial and detrimental inflammatory responses [159].

Several findings highlight the therapeutic potential of GP extracts in mitigating neuroinflammation, offering promising avenues for further research and clinical development. Interestingly, GP extract has been shown to dose-dependently downregulate mRNA levels of pro-inflammatory factors (e.g., TNF-α, TLR-4, IL-1β, iba-1, and iNOS) in LPS-stimulated N13 cells [122]. Additionally, Parekh et al. demonstrated that intragastric delivery of GP extract via nutriosomes significantly protected nigrostriatal dopaminergic neurons in a Parkinson’s disease mouse model [123].

To enhance the therapeutic potential of GP extracts innovative approaches have further exploited. Antioxidant nanoplatforms using polyphenol-rich GP extracts, loaded into functionalized liposomes, showed exceptional neuroprotective efficacy. These nano formulations successfully crossed the blood–brain barrier in vitro, restored ROS levels, prevented α-synuclein fibril aggregation, and improved cell viability in an in vitro rotenone induced PD model [125].

Moreover, inflammation has been implicated in the pathophysiology of migraine, where brain hypersensitivity and neurogenic inflammation play a central role. Calcitonin gene-related peptide, a key inflammatory mediator in migraines, was significantly reduced in a dose-dependent manner by GP extract treatment of neuroendocrine CA77 cells [126].

The potential anti-inflammatory activity of aqueous extracts of red Primitivo pomace was assessed in cultured human endothelial cells exposed to the proinflammatory cytokine TNF-α. It is well known that inflammatory and atherosclerotic stimuli are related to changes in the functional and phenotypic properties of the endothelium; with an overexpression of leukocyte adhesion molecules, such as VCAM-1 and ICAM-1. The GP extract showed powerful inhibition of this processes [127].

Polyphenols present in wine pomaces demonstrated protective actions against renal fibrosis. A key feature of renal fibrosis is epithelial–mesenchymal transition (EMT), which can be triggered by various factors, including reactive oxygen species (ROS) and pro-inflammatory cytokines. During EMT, renal cells undergo a phenotypic transformation, losing their epithelial characteristics and acquiring mesenchymal traits, leading to a subsequent loss of normal epithelial function [160]. The bioavailability of phenolic compounds could improve renal tissue regeneration and prevent renal fibrosis by preserving differentiated epithelial phenotypes under normal and pathological conditions [129].

The profiles of major flavonoid classes present in the red grape skin of Sangiovese red wines have been studied, along with their bioavailability and biological activities for potential cosmeceutical applications. Specifically, an innovative, eco-friendly formulation based on polyphenols extracted using NaDESs was developed. These extracts were further investigated in 3D human keratinocytes (HaCat spheroids) injured by the pro-oxidant agent menadione. The GP extracts demonstrated significant intracellular antioxidant activity and notably reduced the release of the pro-inflammatory cytokine IL-8, thereby enhancing cell viability [90].

Pop et al. demonstrated the cytocompatibility and anti-inflammatory properties of red and white GP extracts in gingival fibroblasts. The extracts reduced the levels of proinflammatory cytokines (IL-6, IL-8, and IL-1β) in a dose-dependent manner. At the highest tested dose of 300 µg/mL, all extracts displayed similar anti-inflammatory effects, decreasing the level of IL-8 by approximately 60% [130].

Properties of hydroethanolic extracts prepared from Pinot Noir GP were evaluated in various conditions. The formulation proposed in this study containing fucoidan-coated silica could be used in nutraceutical or cosmetics due to its anti-inflammatory radical scavenging capacity, while PN@Diatomitematerial could be useful as fertilizer for plants [131].

In summary, GP extracts exhibit significant potential as a sustainable source of bioactive compounds with notable antioxidant and anti-inflammatory properties. The diverse applications of these extracts in the management of oxidative stress-related pathologies and inflammatory disorders are considerable. As our understanding of the intricate mechanisms underlying oxidative stress and inflammation in various pathophysiological states continues to evolve, GP extracts emerge as a valuable addition to our therapeutic armamentarium. These extracts offer a natural, potentially pleiotropic approach to improving human health, warranting continued research to fully elucidate their mechanisms of action, optimize their formulation, and validate their efficacy in clinical settings.

### 5.3. Antimicrobial Activity

Antimicrobial activity denotes the capacity of a substance to inhibit the proliferation or eliminate microorganisms, including bacteria, fungi, viruses, and protozoa.

GP is a rich source of bioactive compounds with demonstrated efficacy against a broad spectrum of human pathogens. A very recent study demonstrated the antibacterial effects of polyphenolic extracts from Aglianico GP against the most common causative agents of food infections that are both Gram-positive, such as *Staphylococcus aureus* and *Bacillus cereus*, and Gram-negative, such as *Escherichia coli* and *Salmonella enterica* [132].

Costa et al. investigated a GP extract derived by the action of an enzymatic cocktail with xylanase activity and subsequently exposed to simulated gastrointestinal digestion to evaluate its stability. This study provides the characterization of its chemical composition and the evaluation of its antimicrobial, prebiotic, and antioxidant activities. It was found to contain high levels of dietary fiber and other carbohydrates, including xylo-oligosaccharides, as well as minerals and phenolic compounds. In vitro simulated gastrointestinal digestion showed that xylobiose was resistant to gastric conditions, whereas phenolic compounds were not. The extract, when used at 2% (*w*/*v*), served as a potential carbon source that could be fermented by *Lactobacillus* and *Bifidobacterium* spp. even after digestion. Additionally, the extract demonstrated significant antioxidant and antimicrobial activities against *Staphylococcus aureus*, *Escherichia coli*, and *Pseudomonas aeruginosa* [133]. In a subsequent study, the same authors evaluated the antimicrobial activity of encapsulated GP extract against pathogenic microorganisms responsible for gastrointestinal disorders. The growth inhibition of methicillin-susceptible *Staphylococcus aureus* (MSSA), *L. monocytogenes*, *P. aeruginosa*, and *S. enteritidis*, and to a lesser extent *E. coli*, was observed. Interestingly, *Candida albicans* was the most sensitive strain, showing a 6-log reduction of viable cell numbers [134]. *Candida albicans* is an opportunistic pathogen and a commensal of the human microbiota, inhabiting the oral cavity, gastrointestinal tract, and female genital tract. Its pathogenicity is closely linked to its ability to form biofilms, structured cell communities encased in a protective extracellular polymeric matrix. Among bioactive phenolic compounds in GP extracts, pterostilbene is a highly effective antifungal, demonstrating greater activity than resveratrol and viniferins. It has been demonstrated the efficacy of pterostilbene and crude extracts from non-fermented GP, encapsulated in poly(lactic-co-glycolic) acid nanoparticles, against *Candida* biofilms [135].

Gomez-Mejia et al. established that grape seed’ extracts from *Vitis vinifera* L. var. Albariño proved to be active against the above-mentioned MSSA, attaining a MIC of 5 mg/mL, followed by *E. faecalis*, *E. coli*, and *P. mirabilis* and finally by *L. monocytogenes*, *P. aeruginosa*, *L. gonorrhea*, and *C. albicans*. It is important to note that seed extracts exhibited high antimicrobial activity and no cytotoxicity against tumor and non-tumor primary liver cells [136].

In addition, it has been reported that white and red GP were able to inhibit *P. gingivalis*, *S. aureus*, *E. coli*, *Klebsiella* spp., and *Citrobacter* spp. [130]. The impact of procyanidin-rich grape seed extract on oxidative stress induced by bacterial lipopolysaccharide (LPS) and the integrity of the epithelial barrier was investigated using a Caco-2 cell model. The extract’s effectiveness in inhibiting biofilm formation was assessed against two major foodborne Gram-negative pathogens, *Salmonella Typhimurium* and *Escherichia coli*. The findings revealed that the extract can modulate critical virulence factors of Gram-negative bacteria, indicating its potential use as an alternative therapeutic approach for maintaining gastrointestinal health [137].

A very recent study explored whether Sangiovese GP extracts exhibit antiviral properties. Very low cell toxicity and potent antiviral activity against *Herpes Simplex virus type-2*, *Feline Calicivirus*, *Coxsackievirus B5*, and *Influenza type-A virus* were observed. Administration before or during infection blocked viral replication completely, demonstrating that GP extracts possess broad-spectrum virucidal action [138].

The antiviral properties of Taurisolo^®^, a novel nutraceutical formulation derived from grape pomace polyphenolic extract, as previously described, were evaluated against *Herpes* virus infections. The authors demonstrated a significant reduction in viral infection rates. Two prominent members of the *Herpesviridae* family (HSV-1 and HSV-2) were chosen. They are widely responsible for recurrent global infections. HSV-1 is predominantly associated with oral herpes transmitted via oral-to-oral contact, whereas HSV-2 is primarily linked to genital herpes, transmitted sexually. Taurisolo^®^ exhibited activity against both HSV-1 and HSV-2. In contrast, it showed no effect against the non-enveloped poliovirus, suggesting that the antiviral mechanism may target the viral envelope [139].

To sum up, the antimicrobial efficacy of GP extracts is primarily attributed to their rich polyphenolic profile, with specific compounds such as pterostilbene demonstrating notable antifungal activity, particularly against opportunistic pathogens like *Candida albicans*. The observed broad-spectrum antimicrobial activity against various microorganisms, including foodborne pathogens, oral microbiota, and enveloped viruses such as *Herpes Simplex* virus types 1 and 2, underscores the potential utility of these extracts in diverse applications, ranging from food preservation to oral hygiene maintenance and antiviral therapeutics.

In the context of the growing global health concern surrounding antimicrobial resistance, these naturally-derived compounds present a promising alternative or adjunctive approach to conventional antimicrobial strategies. However, the translation of these preclinical findings into clinically relevant interventions necessitates further comprehensive research to address the identified knowledge gaps and methodological challenges. This rigorous scientific investigation is crucial for fully elucidating and harnessing the antimicrobial potential of GP extracts in practical applications.

### 5.4. Antiproliferative Activity

Cancer continues to be one of the leading causes of mortality globally, despite significant advancements in both basic research and clinical practice. Early detection and chemoprevention are crucial strategies for reducing cancer incidence. Moreover, the adverse effects associated with conventional treatments often reduce patients’ quality of life, highlighting the need for safer and more effective therapeutic alternatives. Although extensive research has explored natural therapies to combat cancer, not fully satisfactory or comprehensive therapeutic solution has yet been identified [161].

An emerging body of literature increasingly highlights the potential antiproliferative activity GP extracts, primarily attributed to their high flavonoid content, as well as their richness in dietary fiber (DF). This phenol-rich DF matrix functions as a dietary supplement, offering a combination of fiber and antioxidant benefits that contribute to cancer prevention. A comprehensive review published in 2018 further emphasized the anticancer properties of GP fibers, along with its other biological activities [162].

Among the first studies on the antitumor potential of GP extracts, the inhibition of EGFR expression in cutaneous squamous cell carcinoma of the head and neck and of MEPK/ERK1-2 and MAPK/p38 in breast carcinoma was demonstrated, counteracting tumor invasiveness and progression [140]. The antiproliferative properties of GP extract have been corroborated in breast cancer cell studies conducted by other researchers. Polyphenolic compounds derived from *Muscadinia rotundifolia* ‘Noble’ and *Vitis vinifera* ‘Cabernet Sauvignon’ have demonstrated the ability to inhibit the proliferation of breast cancer cell lines MDA-MB-231 and MCF-7. These inhibitory effects were linked to the induction of cell cycle arrest and apoptosis, as evidenced by the downregulation of cyclin A, the upregulation of p21, and the activation of the caspase cascade [141].

Di Meo et al. investigated the anticancer activity of semi-polar extracts derived from grape seeds of two Italian grape varieties, Aglianico and Falanghina. Their study evaluated the extracts’ ability to decrease the growth and migration of three distinct mesothelioma cell lines through the induction of apoptosis [142].

A study investigated the antioxidant and pro-oxidant activities of polyphenol extracts from Syrah and Chardonnay GP on melanoma cells. Treatment with extracts at concentrations of ≥250 μg/mL for 24 h resulted in a 25 to 50% reduction in cell viability compared to controls, with variations depending on the treatment duration, dose, and type of extract. Additionally, the potential of these extracts to inhibit the metastatic progression of B16F10 cells from the primary tumor site was evaluated [143].

In human hepatoma cell line Hep-G2, the incubation with polyphenols obtained from Nero d’Avola GP showed a significant effect on cell inhibition, even at a concentration of 10 μg/mL [111]. Other studies support the activity of GP extract against Hep-G2 cells. In particular, Xia et al., demonstrated the highest activity for the European grapes with respect to Oriental and American grapes [144].

Several studies documented the potential anticancer effects of GP extracts on colon adenocarcinoma cell growth. It has been described that the purified extract (100 μg/mL) inhibited the proliferation of Caco-2 cells by 52.1% at 48 h. Interestingly, other polyphenol-rich foods, e.g., olive oil (100 μg/mL), red wine (100 μg/mL), and a tomato extract (2.5 mg/mL), inhibited cancer cell growth over a similar time [145].

The potential of red GP-based condiments as chemo-preventive agents in colorectal cancer were studied. Notably, the antiproliferative and antigenotoxic effects of these products were demonstrated in the HT-29 cell line. This cell line serves as a widely accepted model for transformed neoplastic colorectal cells, commonly used in studies investigating cell cycle dynamics and genotoxicity [146,147].

Several studies have explored the phenolic composition and biological activity of grape pomace extracts. Tapia et al. identified 35 phenolic compounds in hydroalcoholic extracts derived from the pomace of *Malbec* and *Torrontés* wines from Argentina’s Calchaquí Valleys. Both extracts displayed significant antioxidant and antiproliferative activities. Notably, the *Torrontés* pomace extract was particularly effective against colon cancer cells, reducing cell viability in a dose-dependent manner to 67% in HT-29 cells and 48% in Caco-2 cells [148]. Additionally, Mišković Špoljarić et al. investigated the antiproliferative effects of two phenolic-rich GP extracts on Caco-2 and SW620 colorectal cancer cell lines. Their findings demonstrated a significant reduction in cell growth and disruption of cell cycle progression in these cells [149]. Caponio et al. further examined the mechanisms underlying the antiproliferative activity of gastrointestinal-digested *Aglianico* GP extracts on HT-29 and Caco-2 cell lines. They reported a pronounced ability of the extract to inhibit cell proliferation and induce apoptosis. This was evidenced by a significant increase in the Bax/Bcl-2 ratio and enhanced caspase-3 activity [150].

Using gas chromatography-mass spectrometry-based metabolomic analysis, researchers characterized metabolic shifts in the colorectal cancer-prone mouse model. Mice were selected for this investigation due to their genetic and physiological relevance to human cancer research. The findings demonstrate that dietary GP exerts a protective effect against cancer development. This protective role is supported by specific metabolomic changes, including increased fecal deoxycholic acid, decreased ω-muricholic acid, and reduced amino acid levels and urease activity. These alterations are indicative of improved gut health, metabolic modulation, and potential systemic benefits conferred by GP supplementation. Furthermore, compared to mice not receiving GP supplementation, GP-fed mice exhibited reduced levels of fecal genotoxic metabolites. This reduction was associated with decreased DNA damage and upregulation of the DNA repair enzyme MutS homolog 2 (MSH2), a key component of the DNA mismatch repair pathway [151].

Many comparative studies have aimed to demonstrate antiproliferative activity, often linked to antioxidant and anti-inflammatory effects, across two or more cell lines, including both tumor and non-tumor cells with varying degrees of differentiation. A detailed account of these studies is provided below.

The antitumoral effects of GP extracts from white and red pomace were tested on two colon cancer cell lines and on fibroblasts. The cytotoxic activity demonstrated specificity for colorectal cancer cells, with minimal impact on fibroblasts. Furthermore, the biological activity was primarily attributed to the flavonol and flavan-3-ol subfractions, rather than the anthocyanin subfraction. Preliminary investigations into the mechanisms underlying these effects indicated a downregulation of Myc gene expression. Myc, well-known as an oncogene overexpressed in various human tumors for over two decades, is a transcription factor that, in association with the MAX protein, regulates genes involved in cell cycle progression, angiogenesis, apoptosis, and DNA damage response [163]. The observed reduction in Myc expression suggests a potential antiproliferative effect of the extracts, specifically targeting colorectal tumor cells, as this effect was not observed in fibroblasts [152].

In 2020, the antiproliferative properties of Fetească neagră and Pinot Noir GP extracts were evaluated on human lung carcinoma A549, human breast adenocarcinoma MDA-MB-231, murine melanoma B164A5, and keratinocyte HaCat cell lines. Again, the malignant cells’ proliferation was inhibited by all GP extracts but not that of the normal cells [114].

Ponticelli et al. evaluated the antiproliferative activity of Aglianico GP extracts on hepatoma cells Hep-G2 and on hepatocytes IHH. Their results demonstrated high cytotoxicity against Hep-G2 cancer cells, exerting a pro-oxidant effect, sparing non-tumor hepatic cells [153].

GP extracts obtained from the Croatina variety were administered to cancerous and non-cancerous cell lines. The different cells appear to metabolize GPE compounds differently. Human cervix adenocarcinoma HeLa cells were the most susceptible to GP extract presence, followed by human colorectal Caco-2 cells. On the other hand, the non-cancerous cell lines, human embryonic kidney HEK293T cells and mouse fibroblasts L929, had the highest tolerance. This result shows that for the same GPE concentration, the compounds elicit a toxic effect on the cancerous cell lines, but not on non-cancerous cell lines, opening the door to natural anticancer therapies [154]. Peixoto et al. reported similar results, where GP extracts were only effective against human breast adenocarcinoma MCF7 cells and cervical carcinoma HeLa cells, not showing toxicity for liver non-cancerous PLP2 cells [51].

The anticancer potential of GP extracts was also assessed on mammary and pulmonary-derived types, alongside normal human fibroblasts by Pop et al. The MDA-kb2 cell line showed the highest cytotoxic response to the tested extracts, whereas the A549 and T47D-KBluc cell lines exhibited only moderate anticancer activity. The greater toxicity toward cancer cells has been attributed to disruptions in mitochondrial membrane potential, ultimately leading to apoptosis in these cells. Purified GP extracts affected fibroblast viability to some extent, but their relative potencies were very different from those observed in colorectal cancer cells [130].

Panic et al. investigated the antiproliferative and antioxidative activity of NaDES extracts from GP on HeLa and MCF-7 cancer cell lines. The treatment of HeLa cells with NaDES-based extracts significantly altered the cell cycle distribution, increasing the proportion of cells in the G0/G1 phase and reducing the number of cells in the S phase compared to the control group [155].

Very recently, the antitumor effects of a Sicilian grape pomace (GP) extract were evaluated, comparing two different cell models: HCT116 colon cancer cells and MDA-MB-231 breast cancer cells. The characterization of the extract revealed a high concentration of anthoxanthins and phenolic acids. The extract was more effective in reducing the viability of colon cancer cells than breast cancer cells, which exhibited resistance to the treatment. Specifically, colon cancer cells underwent apoptosis, as evidenced by DNA fragmentation, caspase-3 activation, and PARP1 degradation, while breast cancer cells did not show signs of apoptosis. Nevertheless, autophagy was implicated in both cell lines treated with the extract, as indicated by an increase in acidic vacuoles and elevated levels of p62 and LC3II [156].

The exploration of GP extracts in cancer treatment is compelling and underscores the increasing interest in natural compounds as complementary or alternative therapeutic agents. The presented evidence indicates that GP extracts exhibit notable bioactive properties, justifying further investigation. However, their immediate clinical application remains unfeasible due to the absence of in vivo studies and human clinical trial data. Although in vitro findings are encouraging, they frequently fail to accurately predict therapeutic efficacy in complex biological systems. Furthermore, it is essential to assess potential adverse effects and interactions with existing treatments, as natural compounds are not inherently safe. Nonetheless, research in this field is promising and rigorous clinical trials are necessary to determine whether GP extracts could serve as effective adjuncts in cancer therapy.

## 6. Use of *Vitis vinifera* L. Pomace in Food, Food Supplements and Cosmetics

### 6.1. Food and Food Supplements

The multitude of nutrients and nutraceuticals previously described triggers new perspectives for the rational use of GP in the food industry. The high content of phenolic acids and polyphenol compounds is responsible for GP’s strong antioxidant properties. It can be used for fortifying food considering the content of minerals, vitamins, and especially dietary fiber. Moreover, wine pomace is endowed with antimicrobial activity due to presence of polyphenols. Therefore, pomace not only serves as a fortifying agent but is also capable of slowing down oxidative processes in the product and regulating its rheological properties [164]. This permits the use of GP in food technology and specifically in the technology of flour products, as reported in numerous recent studies and briefly summarized in a mini review [165]. The quantity of wine pomace powder that can be added to food products is typically 5–10% of the total weight, because a lower dosage may not provide adequate enrichment of the product, while a higher dosage can often negatively affect the structural–mechanical and organoleptic properties of the product. The addition of wine pomace powder leads to noteworthy effects in some technological and functional properties of semi- and end-products: (i) improvement of the rheological properties of bread dough; (ii) effects on textural characteristics such as firmness, chewiness, springiness, and cohesion, and (iii) an increase in antioxidant properties for muffins and cookies, consequently reducing lipid oxidation during shelf life. 

A brand new review [166] summarizes and discusses the large number of publications currently to the use of GP in food production (bakery products, meat, beverages, etc.), highlighting its role as a sustainable and functional ingredient with applications in the food and nutraceutical industries, according to the healthy and circular economy perspectives.

Recent studies have explored the protective effects of a polyphenol-rich grape pomace and blueberry extract (Memophenol™) on the microbiota–gut–brain axis in a model of chronic low-grade inflammation. Additionally, a single dose of this polyphenol-rich extract has been shown to enhance cognitive performance in healthy young adults during prolonged cognitive tasks [167].

Several studies concern also the potential use of pomace in the feed of farm animals on the basis of its antioxidant properties, oxidative stress being a common challenge in farm animals. Recent research suggests that dietary supplementation with GP can help mitigating oxidative stress while enhancing testis development and sperm quality in lambs [118,119]. A very recent review summarized the current applications and challenges of using grape by-products from the agro-industrial sector in pig and poultry diets, aiming at improving meat quality and nutritional properties [168]. Harikrishnan and colleagues demonstrated that GP is a very rich source of bioactive nutritional components that could be used in the formulation of diets for the aquatic animal feed industry [117].

Despite the numerous advantages associated with the use of GP, it is important to underline the potential toxicological risks. Lopez and coworkers discussed the topic of the potential presence of mycotoxins in by-products, showing also the presence of ochratoxin in samples of GP [169]. Therefore, another point that must be taken into consideration is the importance of implementation of strict, efficient strategies that reduce mold growth, as well as of monitoring strategies and hygienic precautions against mycotoxins during by-products’ storage [170]. As previously discussed, the tannins in GP have demonstrated antimicrobial properties, inhibiting the growth of certain bacteria and fungi; therefore, they could be useful in counteracting the toxicological risk [171].

### 6.2. Cosmetics

*V. vinifera* L. is a valuable raw material for the cosmetics industry thanks to its many biological properties, including its anti-aging, anti-inflammatory, antioxidant, and UV-protective activities [172,173]. In fact, parts of *V. vinifera* L. (e.g., leaves, seeds, and skin) can be utilized to make cosmetics, according to the CosIng (Cosmetic Ingredients) database: grape extracts can be employed as emollients, humectants, emulsifiers, color additives, or perfumes; seeds as a coloring agent, humectant, or hair- and skin-conditioning agent (Cosmetic Ingredient Database (CosIng): www.ec.europa.eu). Numerous cosmetics based on *V. vinifera* L. such as PhytoCellTech^®^, Caudalie^®^ and Korres^®^ are currently available on the market, with the majority of them being made in China, South Korea, the USA, and southern and central Europe.

Regarding GP, its use as a cosmetic ingredient could contribute to the growth of cosmetic products and, consequently, favor their valorization and provide an effective and environmentally friendly alternative use. Pomaces are abundant in bioactive compounds of cosmetic interest such as polyphenols including phenolic acids (caffeic acid, gallic acid, protocatechuic, 4-hydroxybenzoic, and syringic acid), phenolic alcohols (hydroxytyrosol), flavonoids (quercetin-3-O-rhamnoside, catechin, epicatechin, and luteolin), proanthocyanidins, and stilbenes (resveratrol) [174]. In 2018, Ryu and Na [175] evaluated the cytoprotective activity of syringic acid towards HaCaT cells from damage of UVB radiation. The antioxidant and anti-aging activities of syringic acid render it a potential ingredient in cosmetic products. Hubner et al. [176] evaluated the safety and clinical efficacy of oil in water sunscreen formulations containing a SP extract. The results demonstrated a synergistic effect between the sunscreen system and the bioactive compounds of the extract, mainly anthocyanins, flavanols, and flavonols on UVB protection and antioxidant activity.

Another interesting cosmetic ingredient that can be extracted from GP is gallic acid, which exhibits antioxidant, anti-inflammatory, and also anti-tyrosinase activities, as reported by Ferri et al. [177]. Currently, new tyrosinase inhibitors are being sought to reduce skin pigmentation. One such inhibitor is gallic acid, which, according to Zambrano et al. [178], demonstrated the highest inhibitory activity among all compounds analyzed and may therefore be useful as a skin-whitening ingredient in the cosmetic industry. Using R. miehei NRRL 5282 cellulase, which increases the total phenolic content (TPC) and antioxidant capacity, researchers were able to extract gallic acid and syringic acid from Othello black grapes.

Another polyphenol that is frequently used in cosmetics is resveratrol, which has anti-inflammatory, anti-aging, antimicrobial, and skin-whitening properties. To protect its antioxidant action and make topical use of anti-aging products easier, it can be encapsulated in liposomes or nanoparticles [179].

In addition, GP phenolic compounds have also been studied as ingredients for oral care cosmetics (e.g., oral rinses, toothpastes, dental sprays, or oral hygiene tablets and capsules) thanks to their antimicrobial, anti-odor, anti-inflammatory, astringent, and soothing properties. Procter and Gamble Co. is one of the most prestigious brands of oral care and they have made an association between Citrus and Vitis pulp and seed extracts for a toothpaste with a phenolic composition of nearly 15% [180].

In conclusion, GP represents a valid raw material for cosmetic applications, particularly due to its health benefits, such as its antioxidant, anti-aging, anti-inflammatory, and UV-protection properties. However, while GP offers interesting opportunities for the cosmetic industry, it is imperative to continue research, optimize production processes, and critically evaluate the benefits and challenges associated with its use to ensure safe, effective, and truly sustainable products. Future studies should focus on comprehensive life cycle assessments, long-term clinical trials, and the development of standardized extraction and formulation methods to fully realize the potential of GP in cosmetics.

### 6.3. Patents on the Industrial Application of Grape Pomace

The “WIPO-PATENTSCOPE” world intellectual property database (accessed on 4 January 2025) has been consulted in order to assess the possible industrial uses of grape pomace. Almanza-Oliveros et al. [52] reported patents registered in 2023; thus, we revised this analysis to limit it to 2024, during which 16 patents were registered. In Table 3, the current patents registered in 2024 are reported. Specifically, we have selected the patents in which the use of GP as the main or auxiliary ingredient in the development of beauty and health products is described.

## 7. Conclusions and Future Perspective

Wine pomace, a by-product of winemaking, is increasingly recognized for its diverse applications in the nutritional, pharmaceutical, and cosmetic industries. Rich in bioactive compounds such as phenolic compounds, flavonoids, anthocyanins, and soluble and insoluble fibers, grape pomace (GP) offers numerous health benefits, including antioxidant, anti-inflammatory, and antimicrobial effects. These properties have already led to its incorporation into a variety of commercial products, including nutraceutical supplements, functional foods (e.g., bakery products, protein bars, and functional beverages), and cosmetic formulations like anti-aging creams, shampoos, and exfoliants. Recent research has also revealed its antiproliferative activity, suggesting potential applications in chemoprevention and cancer co-treatment. Furthermore, the presence of additional bioactive components such as vegetal melatonin, phytosterols, essential amino acids, and minerals provides a sustainable, cost-effective means to enhance the intake of these fundamental nutrients and bioactive compounds in consumers’ diets. The valorization of wine pomace not only reduces waste but also supports the development of innovative and sustainable products, contributing to the “3R strategies” of a circular economy: reduce, reuse, and recycle. Its potential applications extend beyond functional foods and cosmetics, with preliminary studies indicating opportunities in regenerative medicine due to its differentiative and osteo-inductive activities.

This review highlights the significant potential of GP for creating value-added products that promote health and well-being while addressing environmental challenges. Moving forward, continued research and technological advancements will be essential to unlock its full potential, transforming wine pomace from an environmental concern into an economic and ecological asset.

## Figures and Tables

**Table 1:** Major phenolic and polyphenolic molecules in GP components (seeds, red skins, and white skins) [49,50,51,52,53,54,55,56,57,58].

Components	Phenolic Compounds	Polyphenols
White Skins	Hydroxycinnamic acids (e.g., caffeic, *p*-coumaric, and ferulic acids)	Flavonols and tannins
Red Skins	Hydroxybenzoic acids (gallic, protocatechuic, syringic, and vanillic acids); stilbenes (resveratrol and piceattanol)	Rich in anthocyanins (cyanidin, malvidin, pelargonidin, peonidin, and petunidin) and flavonols (quercetin, kaempferol, and myricetin)
Seeds	High concentrations of gallic acid, caffeic acid, and protocatechuic acid	Rich in proanthocyanidins (tannins) and catechins

**Table 2:** Overview of grape varieties and corresponding studies on GP extracts, obtained from whole GP (seeds and/or skins), categorized by in vitro, in vivo, and ex-vivo approaches and their associated biological activities. Grape varieties studied, the experimental models employed, and the specific biological activities are reported.

*Vitis vinifera* L. Variety	Type of Study	Biological Activity	Reference Number
Batiki Tyrnavou	In vitro, EA. hy926, C2C12 cells	Antioxidant	[96,97]
Fetească neagră	In vitro and in vivo (Wistar rats)	Antioxidant	[98]
Sour Black, Syrah, Cabernet Franc, Cabernet Sauvignon	In vitro and ex vivo (human platelets)	Antioxidant	[99]
Cagnulari	In vitro, HUVEC cells	Antioxidant	[100]
Pinot noir	In vitro, HUVEC cells	Antioxidant	[101]
Aglianico	In vitro and ex vivo (human neutrophils)	Antioxidant	[102]
Chenin Blanc, Petit Verdot, and Syrah	In vitro, mouse macrophage RAW 264.7	Antioxidant	[103]
Cannonau	In vitro and ex vivo (human erythrocytes)	Antioxidant	[104]
Ruby Cabernet	In vitro and ex vivo (human erythrocytes)	Antioxidant	[105]
Cannonau	In vitro, Caco-2 cells	Antioxidant	[106]
n.s. ^§^	In vitro and in vivo, IPEC-1 cells, crossbred TOPIG hybrid pig	Antioxidant	[107]
Cannonau	In vitro and in vivo, Caco-2, BALB/c mice	Antioxidant	[108]
Cabernet Sauvignon	In vitro, HL-60 cells	Antioxidant, antiproliferative	[109]
Mamaia	In vitro, Hep-G 2 cells	Antioxidant	[110]
Nero d’Avola	In vitro, HS-68, Hep-G2 cells.	Antioxidant, antiproliferative	[111]
Carignano	In vitro, 3T3 cells	Antioxidant	[112]
Cabernet Saugvinon, and Feteasca Neagra	In vitro, HaCat cells	Antioxidant	[113]
Fetească neagră and Pinot noir	In vitro, A549, MDA-MB-231, B164A5, HaCat cells	Antioxidant, anti-inflammatory, antiproliferative	[114]
Croatina and Arneis	In vitro, hMSC cells	Antioxidant, differentiative	[115]
Barbera	In vitro, UMR-106 cells	Differentiative	[116]
n.s. ^§^	In vivo, L. rohita fish	Antioxidant, Anti-inflammatory	[117]
n.s. ^§^	In vivo, ram lambs	Antioxidant,	[118]
n.s. ^§^	In vivo, Hu lambs	Antioxidant,	[119]
Valea Calugareasca,	In vitro, Caco-2 cells	Anti-inflammatory	[120]
Negramaro	In vitro, Caco-2 and HMEC1 cells	Antioxidant, anti-inflammatory	[121]
n.s. ^§^	In vitro, N13 cells	Anti-inflammatory	[122]
Nasco	In vitro e in vivo, Caco-2 cells and C57BL/6J mice	Antioxidant, anti-inflammatory	[123,124]
Albarola, Vermentino, Bosco, Cigliegiolo, Cannaiolo	In vitro, SH-SY5Y cells	Antioxidant, anti-inflammatory	[125]
Tinta cao and Cabernet franc	In vitro, CA77, PC-12 cells	Anti-inflammatory	[126]
Primitivo, Negramaro, Negramaro/Lambrusco	In vitro, HMEC1 and U937 cells	Anti-inflammatory	[127]
Tannat and Cabernet Franc	In vitro, HT-29 cells	Antioxidant, anti-inflammatory	[128]
Tempranillo and Verdejo	In vitro, MDCK cells	Anti-inflammatory	[129]
Sangiovese	In vitro, HaCat cells	Antioxidant, anti-inflammatory	[90]
Pinot Noir, Feteasca neagra, Cabernet Sauvignon, and Mamaia, Muscat Ottonel and Sauvignon Blanc	In vitro, A549, T47D-KBluc, and MDA-kb2	Antioxidant, anti-inflammatory, antimicrobial, antiproliferative	[130]
Pinot Noir	In vitro, RAW 264.7 cells,	Antioxidant, anti-inflammatory antimicrobial	[131]
Aglianico	In vitro	Antimicrobial	[132]
Syrah	In vitro, Caco-2 and HT-29-MTX cells	Antioxidant Antimicrobial	[133,134]
Italia	In vitro	Antimicrobial	[135]
Albariño	In vitro, erythrocytes MCF-7, Hep-G2, NCI-H460, HeLa cells	Antioxidant, antimicrobial	[136]
Bellone	In vitro, Caco-2 cells	Antioxidant Antimicrobial	[137]
Sangiovese	In vitro, VERO E6 MDCK NBL-2, A549 HeLa, CrFK cells	Antimicrobial	[138]
Aglianico	In vitro, Vero cells	Antimicrobial	[139]
n.s. ^§^	In vitro, A431, SCC13, NHEK cells w	Antiproliferative	[140]
Cabernet Sauvignon	In vitro, MDA-MB-231, MCF-7 cells	Antiproliferative	[141]
Aglianico, Falanghina	In vitro, MSTO-221H, NCI-H2452	Antiproliferative	[142]
Syrah, Chardonnay	In vitro, B16F10 cells	Antioxidant, antiproliferative	[143]
Cabernet Sauvignon, Merlot, Muscat Hamburg	In vitro, Hep-G2 cells	Antioxidant, antiproliferative	[144]
Zalema	In vitro, Caco-2	Antiproliferative	[145]
Tempranillo	In vitro, HT-29	Antiproliferative	[146]
Aglianico	In vitro, HT-29	Antioxidant, antiproliferative	[147]
Malbec and Torrontés	In vitro, erythrocytes, Caco-2, HT-29 cells	Antioxidant, antiproliferative	[148]
Cabernet Sauvignon	In vitro, Caco-2 and SW620 cells	Antioxidant, antiproliferative	[149]
Aglianico	In vitro, HT-29, SW480	Antioxidant, antiproliferative	[150]
n.s. ^§^	In vivo, mice	Antiproliferative	[151]
n.s. ^§^	In vitro, Caco-2, HT-29, CRL2072 cells	Antiproliferative	[152]
Aglianico	In vitro, Hep-G2, IHH cells	Antioxidant, antiproliferative	[153]
Croatina	In vitro, L929, HEK293T, Caco-2, HeLa cells	Antioxidant, antiproliferative	[154]
n.s. ^§^	In vitro, HeLa, MCF-7, PLP2 cells	Antioxidant, antimicrobial, antiproliferative	[51]
Plavac mali	In vitro, HeLa and MCF-7 cells	Antioxidant, antiproliferative	[155]
Pinot gris	In vitro, HCT116, MDA MB-231 cells	Antioxidant, antiproliferative	[156]

n.s. ^§^ = not specified variety.

**Table 3:** Current patents in WIPO-PATENTSCOPE database on the industrial application of GP.

Patent Number	Title	Short Abstract	Scope	Publication Date	Country
WO/2024/026416	Botanical extract blend for use in skin care	The invention relates to topical compositions based on muscadine grape pomace and its use for improving the appearance of skin.	Cosmetic product	1 February 2024	USA
RU0002814158	Method for preparing drink from plant materials for healthy diet	The invention describes a method for preparing a drink from grape pomace and hibiscus extracts for a healthy diet.	Food industry	26 February 2024	Russian Federation
CN118160843	Preparation process of pellet feed for sheep breeding	The invention relates to the technical field of animal feed. During the preparation of pellet feed, the use of grape pomace improves the aerobic stability of the feed.	Food technology	11 June 2024	China
WO2024142004	Oleolyte of lyophilized apple or grape pomace with high content of ursolic acid	The invention describes a process for the extraction of ursolic acid (UA) in sunflower oil from lyophilized apple or from lyophilized grape pomace of Fiano di Avellino DOCG variety. Moreover, authors described the oleolyte obtained and the use thereof in cosmetic preparations.	Cosmetic products	4 July 2024	Italy
CN118318939	Production process of concentrated grape juice	The invention discloses to the field of food processing. The concentrated grape juice is produced by different steps and, is mellow in taste and moderate in sourness and sweetness.	Food technology	12 July 2024	China
RO138449	Nutraceutical formulations as uncoated tablets with sustained release for hepato-digestive protection and process for preparing the same	The invention relates to a process for preparing nutraceutical products based on honeyberries, cherries, grape pomace of red grapes, and Lactobacillus probiotics for oral administration, with a hepato-digestive protection effect.	Food	29 November 2024	Romania

## Abbreviations

GP	grape pomace
PUFAs	polyunsaturated fatty acid.
ROS	reactive oxygen species
H2O2	hydrogen peroxide
HOCl	hypochlorous acid
DPPH	2,2-diphenyl-1-picrylhydrazyl
GAE	Gallic Acid Equivalents
AAE	Ascorbic Acid Equivalents
LC-ESI-QTOF-MS/MS	Liquid Chromatography-Electrospray Ionization-Quadrupole Time-of-Flight Mass Spectrometry/Mass Spectrometry
SFE	supercritical fluid extraction
MAE	microwave-assisted extraction
UAE	ultrasound-assisted extraction
EAE	enzyme-assisted extraction
PEF	pulsed electric field
NaDES	natural deep eutectic solvent
HBA	hydrogen-bond acceptors
HBD	hydrogen-bond donors
GSH	glutathione
IBDs	inflammatory bowel diseases
Nrf2	Nuclear Factor Erythroid 2-Related Factor 2
Keap1	Kelch-like ECH-associated Protein 1
TNF-α	Tumor Necrosis Factor-alpha
BMP2	Bone Morphogenetic Protein 2
Runx2	Runt-related transcription factor 2
IFN-γ	Interferon-gamma
IL-12p40	subunit of interleukin-12
IL-7	interleukin-7,
G-CSF	Granulocyte Colony-Stimulating Factor
GM-CSF	Granulocyte-Macrophage Colony-Stimulating Factor
MCP-1	Monocyte Chemoattractant Protein-1
MMP-9	Matrix Metalloproteinase-9
MMP-2	Matrix Metalloproteinase-2
LPS	Lipopolysaccharides
IL-1β	interleukin-1 beta
ICAM-1	Intercellular Adhesion Molecule 1
VCAM-1	Vascular cell adhesion protein 1
COX-2	cyclooxygenase-2
IL-8	interleukin-8
IL-6	interleukin-6
NF-κB	nuclear factor kappa-light-chain-enhancer of activated B cells
TLR-4	Toll-like receptor 4
iNOS	inducible nitric oxide synthase
EMT	epithelial mesenchymal transition
HSV-1	herpes simplex virus type 1
HSV-2	herpes simplex virus type 2
EGFR	epidermal growth factor receptor
PARP1	poly (ADP-ribose) polymerase 1
LC3II	LC3-phosphatidylethanolamine conjugate
